# Feline calicivirus and natural killer cells: A study of its relationship in chronic gingivostomatitis

**DOI:** 10.14202/vetworld.2023.1708-1713

**Published:** 2023-08-24

**Authors:** Ana C. Fontes, Maria C. Vieira, Marcela Oliveira, Lígia Lourenço, Carlos Viegas, Pedro Faísca, Fernanda Seixas, João F. Requicha, Maria A. Pires

**Affiliations:** 1 Department of Veterinary Sciences, University of Trás-os-Montes and Alto Douro, Vila Real, Portugal; 2 Life and Health Sciences Research Institute (ICVS), School of Medicine, University of Minho, Campus Gualtar, 4710-057 Braga, Portugal; 3 ICVS/3B’s-PT Government Associate Laboratory, Braga/Guimarães, Portugal; 4 Animal and Veterinary Research Centre (CECAV) and AL4AnimalS, UTAD, Vila Real, Portugal; 5 Faculty of Veterinary Medicine and Research Centre for Biosciences and Health Technologies, Lusófona University, Lisboa, Portugal; 6 DNAtech, Lisboa, Portugal

**Keywords:** feline calicivirus, feline chronic gingivostomatitis, immunohistochemistry, natural killer cells

## Abstract

**Background and Aims:**

Feline chronic gingivostomatitis (FCGS) is a frequent chronic inflammatory condition in the oral cavity with an etiopathogenesis not completely identified. This study aimed to contribute to the knowledge of FCGS by identifying the presence of feline calicivirus (FCV) antigens and natural killer (NK) cells and comparing them.

**Materials and Methods:**

Forty biopsies from the oral mucosa of cats diagnosed with chronic gingivostomatitis were subjected to immunohistochemical techniques to evaluate cells with FCV antigens and NK cells positive for CD56.

**Results:**

NK cells were identified in all samples, with an average of 725.3 ± 409.1 cells. Regarding FCV, it was identified in 18 out of 30 samples (60%), with a different number of cells with virus in between the analyzed cases. In all cases, the number of cells infected with FCV was lower than the number of NK cells present in the same samples, but there was no statistical association between them.

**Conclusion:**

This preliminary study shows that NK cells are present in gingivostomatitis lesions not exclusively caused by FCV-stimulus, as only 60% of all cases were positive for this virus, but other antigens should be considered in the etiology of FCGS.

## Introduction

Oral cavity diseases are very common in feline practice and present with different clinical presentations on oral examination. Histological evaluation is essential for identification and classification based on the assessment of tissue’s architecture and organization [1, 2]. Feline chronic gingivostomatitis (FCGS) is a frequent chronic inflammatory condition of the oral cavity [1]. Typically, the lesions have a bilateral distribution in the oral cavity and appear more frequently in the glossopalatine arches and fauces (caudal stomatitis) and adjacent to the teeth at the alveolar mucosa (alveolar mucositis); also involving less frequently at the base of the tongue, the rostral region of the oral cavity of the pharynx, palate, and lips [1–3]. From a histological point of view, it is characterized by an oral diffuse inflammatory response that ultimately leads to ulcerative-proliferative lesions due to lymphoplasmacytic cell infiltration [4, 5]. Although its etiology is not completely identified, there may be a relationship between inflammation, associated with an exacerbated immune response, and the occurrence of lesions in the oral cavity [6–8]. Natural killer (NK) cells represent the third population of lymphocytes, after B and T cells, drifting from the same bone marrow precursors. Natural killer lymphocytes are part of the innate immune system, most of them with cytotoxic activity. They produce lytic granules containing an arsenal of molecules (perforin and granzymes) that can induce cell death in the target cells. They also express several tumor necrosis factor superfamily members, which induce target cell apoptosis by binding to their corresponding surface receptors. NK cells produce an array of cytokines, growth factors, and chemokines and can also modulate the immune response by interacting with dendritic cells, macrophages, and T cells [9]. Through these mechanisms, NK cells represent with other innate immune cells, the first line of defense against pathogens such as viruses, bacteria, parasites, or even tumor cells without previous immune stimulation and are linked with the adaptive immune response [10, 11]. On infection, viruses are first recognized by the cells of the innate immune system, triggering an inflammatory response to inhibit its action, through mechanisms such as the release of type I interferons (IFN-alpha(a) and IFN-beta(b)) and the lysis of infected cells by NK cells. In addition to their natural cytolytic action, these cells have a cytotoxic action, in the case of class I major histocompatibility complex decreased expression on the surface of infected cells, which could occur by the virus action [12]. Among other functions, these lymphocytes also have mechanisms that will ultimately lead to cell death of virus-infected cells, namely, by the secretion of lytic granules and death mediated by death receptors [13].

Feline calicivirus (FCV) has been described as a very contagious pathogen and is, therefore, widely distributed in the feline population [14]. Together with feline herpesvirus, *Chlamydophila felis*, and *Bordetella bronchiseptica*, FCV is one of the main infectious agents responsible for the upper respiratory tract of cats [15]. This virus has a small-genome, positive single-stranded RNA virus, without an envelope, with a capsid formed by a single protein. On the surface of this, protein is the region of greatest variability of the virus, the main target for the host’s immune system [16, 17]. Feline calicivirus infection can cause clinical signs of acute upper respiratory and oral diseases, a condition that could be immune-mediated [18, 19]. Recent studies suggest that the FCV infection is important in the progression of oral disease and consequently in triggering the inflammatory process [20].

Based on histopathological samples and immunohistochemistry evaluation, this study aims to contribute to the knowledge of FCGS and the immunological composition of the oral mucosa of diseased animals. Our main objective was to evaluate FCV antigens and NK cells and correlate their presence with the pathological condition of FCGS.

## Materials and Methods

### Ethical approval

All specimens examined were collected for diagnostic purposes as part of standard routine care, based on the best clinical judgment of treating physicians, without causing harm or suffering to the animals or intended to be used for scientific purposes only.

### Study period and location

This study was conducted from January 2021 to June 2022 at the University of Trás-os-Montes e Alto Douro.

### Samples

Forty samples of oral mucosa collected by incisional biopsy from cats with a previous clinical diagnosis of chronic gingivostomatitis were selected from the archives of the Laboratory of Histology and Anatomical Pathology of University of Trás-os-Montes e Alto Douro and DNAtech laboratories. Tissues were fixed in 10% of buffered formalin and embedded in paraffin. Epidemiological and clinical data were collected from the histopathological request forms and medical records.

### Immunohistochemistry protocol

Tissue sections were deparaffinized and hydrated and antigen retrieval was performed in a microwave in citrate buffer, pH 6, for 3 cycles of 5 min in a 750 W microwave. The Novolink™ Max-Polymer detection system (Leica Biosystems, Newcastle, UK) was used for visualization, according to the manufacturer’s instructions. Slides were incubated with CD56 antibody (monoclonal anti-human CD56 antibody, clone 123C3, DakoCytomation) diluted 1:200, and anti-FCV1-43 antibody (mouse monoclonal anti-FCV antibody, ab33990, Abcam) with 1:50 dilution, both overnight at 4°C in a humid chamber.

**Figure-1:**
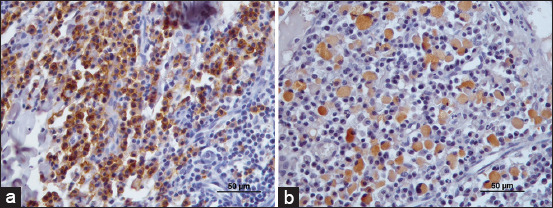
(a) - Immunostaining for CD56 (natural killer [NK]) cells. A strong infiltrate of NK cells is observed in the stoma. (b) - Immunopositivity for feline calicivirus. The positive cells demonstrate a brown cytoplasm. Counterstaining with Gill’s Hematoxylin. Bar=50 μm.

Sections were rinsed with phosphate-buffered saline (PBS) in each technique step. The color was developed with 3.3-diaminobenzidine tetrahydrochloride and sections were counterstained with Gill’s hematoxylin, dehydrated, and mounted with entellan (Figure-1).

As a positive control for the NK identification, a lymph node was used; and as a positive control for the FCV, we used tissue from a polymerase chain reaction (PCR)-calicivirus-positive animal. Negative controls for both NK and FCV were performed by replacing the primary antibody with PBS.

### Positivity assessment, treatments, and data analysis

All slides were independently evaluated by three observers (ACF, MCV, and MAP), in a blind test, and immunopositive cells were counted in five high-power magnification fields (400×). In each case, five photographs were taken at hotspot areas using a Nikon E-600 optical microscope, and images were acquired with the Nikon Imaging Software (NIS) elements D program from Nikon^®^ (Japan). Images were analyzed and anti-CD56 immunopositive cells (CD56^+^) and anti-FCV1-43 immunopositive cells (FCV1-43^+^) were counted, using ImageJ^®^ software (National Institutes of Health, USA).

### Statistical analysis

Microsoft Office Excel spreadsheet (Microsoft, USA) and the statistical analysis program GraphPad Prism, version 8.0 (GraphPad Software, USA) were used for database composition and statistical analysis. Descriptive statistical analysis was performed to characterize the sample according to the variables: Breed, gender, and age (based on American Animal Hospital Association/American Association of Feline Practitioners feline life stage guidelines [21]).

Statistical analysis was performed using non-parametric tests in all analysis, as none of the results presented a normal distribution (Shapiro–Wilk test, p < 0.05). To analyze the influence of clinicopathological parameters on FCV1-43^+^ and CD56^+^ cells, a non-parametric Kruskal–Wallis test was performed separately between groups. To analyze the relationship between FCV1-43^+^ and CD56^+^, Mann–Whitney (MW) test was also performed. Results were statistically significant when p ≤ 0.05.

## Results

### Description of the population

The 40 studied samples were obtained from 16 female (40.0%) and 13 (32.5%) male cats. No data were available for gender in 11 samples (27.5%). These cats were aged between 10 months and 13 years (average of 6 years and 9 months; median 7 years), with this data unknown in 16 animals (40.0%). According to the feline life stages, there was a high frequency of young adult cats (1–6 years old) and adult cats (7–10 years old), both with 9 cases (22.5%), followed by senior cats (aged over 10 years) (n = 5; 12.5%) and finally young cats up to 1 year (n = 1; 2.5%).

Regarding breed, European Shorthair cats predominated (n = 23, 57.5%), followed by the Persians (n = 4, 10.0%) and British Shorthair and Russian Blue cats (both with one case). The breed was not possible to determine in 11 cats (27.5%).

### Assessment of FCV antigens

Positivity for calicivirus antigens was observed in the cytoplasm of infected inflammatory cells (probable macrophages) present in the submucosal connective tissue. In the analysis of the FCV1-43^+^ cell counts, two groups were identified: (i) Group of negative samples, with no labeled cells, (17 cases; 42.5%) and (ii) group of positive samples (23 cases; 57.5%).

**Figure-2:**
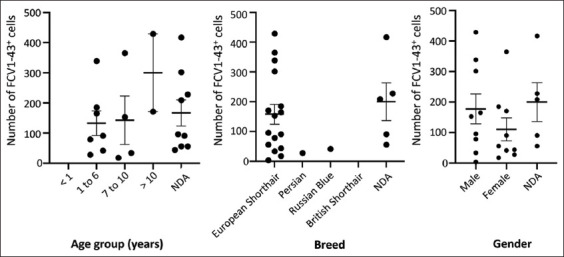
Distribution of the number of FCV1-43^+^ cells according to age group (p = 0.322), breed (p = 0.283), and gender (p = 0.322). Results presented with deviation from the mean of counted cells. NDA=No data available.

Positive cases for the anti-FCV1-43^+^ antibody presented an average of 132.70 ± 40.87 positive cells in young adult cats (1–6 years), 142.50 ± 80.05 cells in adult cats (7–10 years old), and 300.00 ± 129.00 cells in senior cats (over 10 years old). Animals without information about age presented an average of 166.60 ± 43.59 cells per case (Figure-2). No significant association between age and the number of positive cells present in tissues was observed (p > 0.05).

When associating FCV1-43 positivity and breed, the average number of positive cells in the European Shorthair cats was higher (158.20 ± 33.38) than that observed in Persian cats (28 positive cells) or in Russian Blue cats (42 positive cells) (Figure-2). Notwithstanding, no statistically significant association was observed (p > 0.05), noting that some breeds had only one animal.

Regarding gender, although males presented a higher number of FCV1-43 (177.90 ± 48.97) positive cells than females (111.10 ± 37.60) (Figure-2), no statistically significant association was found (p > 0.05).

### Assessment of natural killer cells (CD56^+^)

Positivity was observed as a brown color on the cytoplasm and membrane of cells, scattered on oral tissue samples and on positive control.

**Figure-3:**
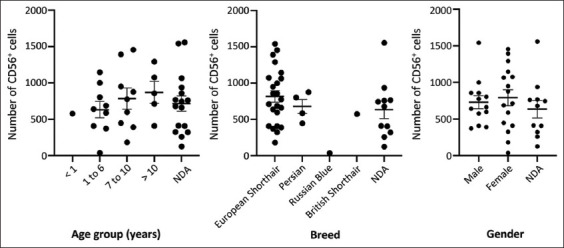
Distribution of the number of CD56^+^ cells according to age group (p = 0.726), breed (p = 0.282), and gender (p = 0.50). Results presented with deviation from the mean. NDA=No data available.

According to the age groups, CD56***^+^*** counts increased with age: There was an average of 575.00 ± 0.00 CD56*^+^*cells in cats up to 1 year old, 631.30 ± 112.10 CD56*^+^*cells in young adult cats (1–6 years), 785.70 ± 145.40 positive cells in adult cats (7–10 years), and 869.20 ± 151.40 positive cells in senior cats (over 10 years of age) (Figure-3). Despite this increase, no significant association between age and NK CD56^+^cells was found (p > 0.05).

Analyzing the CD56^+^ cell counts and breed, the number of NK cells was higher in the common European cats (817.30 ± 80.41) than in Persian cats (679.30 ± 98.88). In the Russian blue breed, only 37.00 ± 0.00 CD56^+^ cells were observed, while in the British Shorthair, 575.00 ± 0.00 positive cells were counted (Figure-3). There was no significant association between cat breed and NK CD56^+^ cells (p > 0.05).

Regarding gender, counts were similar in both male and females: There was an average of 729.40 ± 86.72 positive cells in males and 790.40 ± 108.90 positive cells in females (Figure-3); therefore, no significant association was achieved.

### Correlation between FCV1-43^+^ and CD56^+^

**Figure-4:**
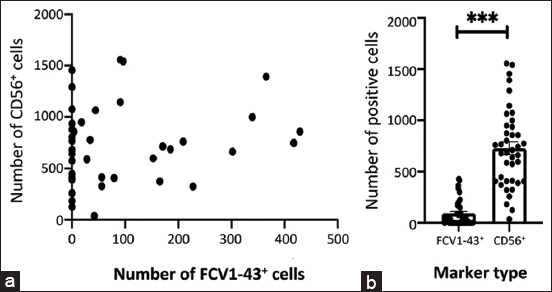
Analysis of the ratio of FCV1-43^+^ and CD56^+^ cell counts. Dispersion of the number of FCV1-43^+^ cells and the number of CD56^+^ cells in each sample (a). Comparison between labeling FCV1-43^+^ cells and labeling CD56^+^ cells (b). (Z(U)=63, p < 0.0001, R=9.9). FCV=Feline calicivirus.

When comparing the association between FCV1-43^+^ and CD56^+^ cells (n = 23), significant statistical differences were achieved between FCV-43^+^ positive cells (mean of 90.05 ± 20.05) and CD56***^+^*** positive cells (mean of 727.60 ± 60.82 positive cells) (MW test: Z(U) = 63, p < 0.0001 two tailed) (Figure-4).

## Discussion

Feline chronic gingivostomatitis is associated with severe inflammation and ulceration in the oral tissues, which persists over time [4]. The multifactorial nature of FCGS is associated with both infectious and non-infectious agents, in particular viral infections which lead to an increase in the T lymphocyte/B lymphocyte ratio [22]. Therefore, the role of several infectious agents in oral inflammation has been studied, namely, feline herpesvirus, feline immunodeficiency virus (FIV), feline leukemia virus, and FCV [23] which led to exploring the role of the latter in the oral inflammation of cats with chronic gingivostomatitis.

In the studied samples of feline diseased oral mucosa, we observed that not all of them showed FCV1-43 positive cells, corroborating other published data. The presence of viral agents in oral lesions was not identified, either by PCR or by IHC [3], but was identified by PCR technique in the oral cavity of cats with clinical signs, as well as in healthy animals [24]. This study demonstrated that the prevalence of FCV in a population of cats in Brazil was higher in healthy cats than in those with clinical signs. As early as 1991, it was reported that cats infected with FCV did not exhibit a higher frequency of FCGS or greater severity of lesions [24, 25]. These controversial results, as well as those in our study, did not show a direct correlation between the presence of this pathogen and the clinical parameters studied.

In some samples, there was no identification of FCV positivity. However, this does not necessarily imply its absence, since the oral biopsies represent only a small portion of the observed oral lesions, which may lead to false negative results, since we do not know how the agent is distributed on the affected tissues [3]. Nevertheless, our positive control as positive results on PCR and in the immunohistochemistry technique on its oral sample gives some confidence to our results.

Although the main goal of these studies was the evaluation and morphological characterization of inflammation, the final purpose was to identify a therapy for this disease. Several treatments over the years have been studied for this disease and even so, there is still no fully effective therapy. Some recommended strategies include rigorous dental cleaning, full-mouth tooth extraction, depending on the severity of clinical signs, antibiotics, corticosteroids, and/or other immunomodulators or immunosuppressive drugs, such as cyclosporine A, chlorambucil, gold salts, or thalidomide [19]. Recently, some studies have shown positive results, in 72% of patients, with the use of mesenchymal stem cells as a viable alternative in the control of T cells [26].

Antivirals used in companion animals only inhibit the replication of DNA viruses or retroviruses and antiviral treatments for FCV infections are not currently a common practice. Ribavirin, one of the few antiviral agents capable of inhibiting the replication of FCV *in vitro*, has proved to be toxic to cats and has side effects, preventing its systemic use [27]. The use of feline IFN-omega (Ω) and human IFN for intralesional or systemic combined with intralesional application has been indicated in studies as effective for treating chronic stomatitis caused by FCV infection [26]. Immunomodulatory treatments are therapeutic methods that are still under development, which is why research into several types of IFN in cat mucosa with FCGS is important. This fact highlights the importance of such studies, as the one presented here, which contribute to the histological and immunological characterization of the oral cavity and, therefore, to the identification of possible therapeutic targets to be explored in the future [28].

Among the various inflammatory populations, we targeted the NK cells (CD56^+^), due to its recognized cytotoxic effect on pathogens, namely, viruses [29, 30]. It is important to note that the CD56 molecule is also a pathogen recognition receptor [31] and that this molecule has been shown to have a functional role in the recognition of other agents, including some fungi [32]. Nevertheless, no histological evidence of fungal agents was found in the samples studied, and in most of these, a considerably high number of NK cells were identified.

Despite the role of NK cells in fighting viral infections, this study did not identify a direct relationship between the presence of FVC and the NK cell counts or distribution in the diseased oral mucosa. These findings go in accordance with Simões *et al*. [33] findings who previously reported no differences between the total number of NK cells in cats infected with FIV in comparison with healthy animals. Moreover, they also suggested that FIV infection may impact the recruitment, death, and/or proliferation of NK cells [33]. An increase in NK cell count along with age without statistical significance was observed, possibly correlated with other unidentified antigen increases in the tissues, or some immunosuppression of the animal. The histological and immunological characterization of the oral cavity, as well as the knowledge of the mechanisms of inflammation and its etiology is fundamental for the understanding of FCGS mechanisms and for the development of new therapies. Further studies are recommended for a better understanding of the pathophysiology of this debilitating feline disease.

## Conclusion

The present study showed that there was no influence of cat’s age, breed, or gender on the presence and frequency of FCV1-43^+^ and CD56^+^cells found in chronic gingivostomatitis lesions.

Several CD56*^+^*NK cells were observed, even when FCV antigens were not identified, suggesting that the presence of NK cells in cats with chronic gingivostomatitis is not exclusively due to the presence of FCV in these oral lesions, but could be related with the presence of other viruses or other antigens confirming the proposal for a multifactorial etiology of FCGS.

Further studies should be performed to clarify the presence of other types of antigens in the etiology and pathogenesis of FCGS, as well as to characterize other inflammatory cell types in this feline morbid process. These studies should include a larger number of samples, with a detailed description of the individuals, including data such as clinical and vaccinal history, as this is one limitation of our retrospective study, where some missing data are not available anymore.

## Authors’ Contributions

ACF, CV, JFR, and MAP: Conception of the study, methodology, supervision, and validation. ACF, MCV, MO, FS, LL, PF, and MAP: Acquisition of data, analysis, and interpretation of data. ACF, FS, CV, JFR, and MAP: Original draft preparation. ACF, JFR, and MAP: Editing of the manuscript. All authors have read, reviewed, and approved the final manuscript.
